# Non-invasive diagnosis of invasive fungal disease with cell-free DNA PCR

**DOI:** 10.1128/jcm.01236-24

**Published:** 2025-10-30

**Authors:** Jordan Kit Mah, Anthony Lieu, Niaz Banaei

**Affiliations:** 1 Department of Pathology, Stanford University School of Medicine 10624 , Stanford, California, USA; 2 Clinical Microbiology Laboratory, Stanford Health Care, Stanford, California, USA; 3 Department of Medicine, Maisonneuve-Rosemont Hospital, Université de Montréal, Québec, Canada; 4 Research Center of Maisonneuve-Rosemont Hospital, Université de Montréal, Québec, Canada; 5 Department of Medicine, Division of Infectious Diseases and Geographic Medicine, Stanford University School of Medicine 10624 , Stanford, California, USA; 6 Department of Medicine, Division of Infectious Diseases, University of British Columbia 8166, Vancouver, British Columbia, Canada; 7 Department of Pathology, University of British Columbia 8166, Vancouver, British Columbia, Canada; Vanderbilt University Medical Center, Nashville, Tennessee, USA

**Keywords:** invasive fungal disease, immunocompromised hosts, cell-free DNA PCR, non-invasive testing

## Abstract

Invasive fungal disease (IFD) is a major cause of morbidity and mortality in the immunocompromised population. Early diagnosis is challenging due to the low sensitivity and non-specificity of non-invasive fungal biomarkers, the need for invasive specimen collection, and the limitations of culture and histopathology. Detection of circulating fungal cell-free DNA (cfDNA) in plasma and serum by polymerase chain reaction (PCR) represents a novel testing modality for rapid and accurate diagnosis of IFD. In this review, we summarize the performance characteristics of fungal cfDNA PCR for the diagnosis of invasive aspergillosis, mucormycosis, and *Pneumocystis* pneumonia. We discuss a testing algorithm that incorporates fungal cfDNA and the added diagnostic value of invasive specimen collection when non-invasive mold cfDNA PCR is performed first. Lastly, we discuss the role of diagnostic stewardship in fungal cfDNA PCR testing.

## INTRODUCTION

Invasive fungal disease (IFD) is a major cause of morbidity and mortality in immunocompromised populations (1). The incidence of IFD over the past decade has increased due to the expanding population of patients on immunosuppressive therapies for various conditions, including organ transplantation, malignancies, autoimmune disorders, and Coronavirus disease 2019 (COVID-19) (2–5). Although early diagnosis and treatment for IFD are associated with lower mortality, accurate diagnosis of IFD is hampered by several challenges (6). First, there is significant overlap in the radiographic and clinical presentations of IFD caused by various molds with different treatments, thus making laboratory diagnosis essential. Second, conventional fungal diagnostics, such as fungal culture and histopathology, are insensitive and nonspecific, respectively, and often require the collection of an invasive specimen through bronchoscopy and biopsy. However, only a fraction of patients with suspected IFD can undergo an invasive procedure due to clinical instability, the presence of thrombocytopenia, or coagulopathy (7). Third, when fungal culture is positive, it usually takes days to weeks for the laboratory to isolate and identify the organism. Lastly, the available non-invasive fungal biomarkers, such as serum *Aspergillus* galactomannan (GM) and 1-3-ß-D-Glucan (BDG), lack sensitivity and/or specificity for the diagnosis of IFD, thus limiting their utility in clinical practice for identifying the exact fungal etiology (8, 9). Because of these diagnostic challenges, accurate diagnosis of IFD is often delayed, which contributes to delayed initiation of optimal antifungal therapy and often results in exposure of patients to unnecessary antimicrobials and their associated toxicities.

Detection of circulating cell-free DNA (cfDNA) fragments in plasma and serum, also colloquially referred to as “liquid biopsy,” is a novel non-invasive testing modality for rapid diagnosis of various diseases and holds great promise for the diagnosis of infectious diseases that currently require an invasive procedure to collect a diagnostic specimen. Fungal cfDNA PCR on plasma and serum was implemented by European microbiologists more than a decade ago and has shown promising results for accurate diagnosis of IFD (10, 11). In this review, we summarize the performance characteristics of fungal cfDNA PCR on plasma and serum for the diagnosis of invasive aspergillosis (IA), mucormycosis, and *Pneumocystis* pneumonia. When possible, we compare the accuracy of cfDNA PCR on plasma and serum to conventional non-invasive serum fungal biomarkers such as GM and BDG. We also discuss a diagnostic algorithm incorporating fungal cfDNA PCR on plasma and the added value of invasive specimen collection when non-invasive cfDNA PCR precedes it. Lastly, we discuss the role of diagnostic stewardship in fungal cfDNA PCR testing.

## PRE-ANALYTICAL FACTORS

Two studies investigating the impact of pre-analytical variables on the detection of fungal cell-free DNA have shown that plasma is superior to serum in sensitivity (91% vs. 80% and 95% vs. 68%, respectively) for the detection of *Aspergillus* cfDNA, which may be due to loss of cfDNA in the blood clots (10, 12). However, it remains to be shown whether this is true for other molds, such as Mucorales agents, and whether it is dependent on the extraction method (10, 12–14). For downstream PCR testing, the commonly used K2 ethylenediaminetetraacetic acid blood collection tube has been shown to be significantly more sensitive for recovery of microbial cfDNA compared with dedicated cfDNA blood collection tubes (15). Once blood is collected, processing for plasma collection can be delayed at least 24 h without any impact on the detection of cfDNA by PCR (15). A single low-speed spin (500× *g* for 10 min) plasma separation from the cellular fraction has been shown to be sufficient (15). Based on spiking experiments, plasma can be stored at −80°C for long-term storage without loss of cfDNA viability (15). The volume of plasma or serum used to extract nucleic acids has been shown to be a critical factor for the downstream detection of microbial cfDNA (15–17). Published studies report 4 mL of plasma in adults as the optimized volume and 1 mL as the minimum volume in pediatric patients (13–21). Lastly, commercial nucleic acid extractors have been shown to be biased toward longer length and more abundant cfDNA fragments, such as human cfDNA, with some methods performing better than others for shorter and less abundant microbial cfDNA (21). Further research is needed to determine the size distribution of fungal cfDNA and to determine whether fungal cfDNA is mostly naked or if it is membrane-bound in extracellular vesicles (22). This type of knowledge may allow developers to optimize the extraction of fungal cfDNA and improve the diagnostic sensitivity of fungal cfDNA PCR. Furthermore, studies are needed to decipher the role of the patient’s immune status and the fungal species in the generation and turnover of detectable cfDNA in blood. Prior studies on mucormycosis have shown that immunosuppression is a determinant of higher Mucorales cfDNA PCR sensitivity, but such an association was not observed in patients with coccidioidomycosis (Lalljie et al., unpublished data) (23, 24). Thus, sensitive detection of fungal cfDNA with PCR for diagnosing IFD likely depends on the patient’s immune status and the fungal species.

## ANALYTICAL FACTORS

Many laboratory-developed PCRs have been reported, and several non-FDA-approved commercial assays are also available for the detection of fungi causing IFD (Tables 1 and 2). Important considerations for sensitive detection of cfDNA include selecting PCR assays that target multicopy sequences, such as the ribosomal RNA genes, and choosing short amplicons (<100 bp) given that bacterial and human cfDNA in plasma have been shown to consist of short fragments (25, 26).

**TABLE 1:** Clinical and assay characteristics and performance of *Aspergillus* cell-free DNA PCR reported in published studies^*a*^

Study, Year (reference)	Patient population	EORTC/MSGERC Category for IMD cases	Number of cases (category) / controls	Specimen type	Sample volume	Extraction method	PCR assay (LDT vs commercial)	Sensitivity % (95% CI, n/N)	Specificity % (95% CI, n/N)
Florent et al. 2006 (27)	Screening for IMD in high-risk patients with HM and HSCT	Proven or probable	33 (4 proven, 29 probable) / 116	Serum	0.2 mL	QIAamp DNA Mini Kit (Qiagen)	LDT	87.9 (71.8–96.6, 29/33)	55.2 (45.7–64.4, 64/116)
El Mahallawy et al. 2006 (28)	Suspected IMD in high-risk pediatric malignancies	Proven or probable	28 (15 proven, 13 probable) / 49	Serum	NP	QIAmp DNA Mini Kit and QIAmp Blood Mini Kit (Qiagen)	LDT	75.0 (55.1–89.3, 21/28)	91.8 (80.4–97.7, 45/49)
Suarez et al. 2008 (17)	Screening 1×/wk for IMD in HM and BMT	Proven or probable	17 (1 proven, 14 probable, 2 possible) / 107	Serum	1 mL	MagNA Pure LC Total Nucleic Acid Isolation Kit (Roche)	LDT	100 (90.5–100, 17/17)	96.7 (92.1–98.3, 104/107)
Lopes da Silva et al. 2010 (29)	Screening 2×/wk for IMD in high-risk patients with HM	Proven or probable	20 (2 proven, 18 probable) / 173	Serum	NP	DNAzol BD reagent (Invitrogen)	LDT	75.0 (50.6–90.4, 15/20)	91.9 (86.5–95.3, 159/173)
Springer et al. 2013 (30)	Screening 2×/wk for IMD in patients with HM and HSCT	Proven or probable	47 (7 proven, 40 probable) / 31	Serum	1 mL	QIAamp UltraSens virus kit (Qiagen)	LDT (WB PCR)	85.1 (72.3–92.6, 40/47)	64.5 (47.0–78.9, 20/31)
					0.5 mL	High Pure PCR template preparation kit (Roche)	LDT Serum PCR	78.7 (65.1–88.0, 37/47)	83.9 (67.4–92.9, 26/31)
Schwaringer et al. 2013 (31)	Screening 2×/wk for IMD in high-risk patients with HM	Proven or probable	11 (2 proven, 9 probable) / 174	Serum	1 mL	MagNA Pure LC DNA (Roche)	LDT	72.7 (39.0–94.0, 8/11)	45.4 (37.9–53.1, 79/174)
Aslan et al. 2015 (32)	Suspected IMD in HM with neutropenic fever	Proven, probable, or possible	78 (1 proven, 17 probable) / 60 possible) / 83	Serum	0.2 mL	QIAamp DNA mini kit (Qiagen)	Commercial (Myconostica MycAssay Aspergillus)	65.4 (58.0–72.7, 51/78)	57.8 (50.2–65.5, 48/83)
Bellanger et al. 2015 (33)	Screening 2×/wk for IMD in patients with HM	Proven or probable	30 (2 proven, 28 probable) / 154	Serum	1 mL	MagNa Pure Nucleic Acid Iso-	LDT	50.0 (31.3–68.7, 15/30)	84.4 (77.7–89.8, 130/154)
						lation Kit (Roche)			
Pini et al. 2015 (34)	Suspected IMD in patients with HM and non-HM	Proven or probable	30 (2 proven, 28 probable) / 41	Serum	0.5 mL	High Pure PCR Template Preparation Kit (Roche)	Commercial (Myconostica MycAssay Aspergillus)	Overall	
								46.7 (28.3–65.7, 14/30)	97.6 (87.4-– 99.9, 40/41)
								HM	
								60.0% (32.3–83.7, 9/15)	97.6 (87.4–99.9, 40/41)
White et al. 2015 (10)	Case control study of plasma vs. serum	Proven or probable	19 (1 proven, 18 probable) / 42	Serum and occasionally plasma	0.5 or 1 mL	DSP virus kit (Roche) and QIAamp UltraSens virus kit (Qiagen)	LDT	68.4 (46.0–84.6, 13/19)	76.2 (61.5–86.5, 32/42)
				Plasma				94.7 (75.4–99.1, 18/19)	83.3 (69.4–91.7, 35/42)
Boluk et al. 2016 (35)	Suspected IMD in patients with HM	Proven or probable	40 (6 proven, 34 probable) / 13	Serum	NP	ZR Fungal/Bacterial DNA	Commercial (Way2 Gene Fungi)	90.0 (76.3–97.2, 36/40)	73.3 (46.2–95.0,10/13)
						MiniPrep Kit (Zymo)			
Springer et al. 2016 (36)	Screening 2×/wk for IMD in patients with HM and HSCT	Probable	18/151	Serum	1 mL	QIAamp UltraSens virus kit (Qiagen)	LDT	Overall	
								94.4 (72.7–99.9, 17/18)	51.0 (42.7–59.2, 77/151)
								Mold Prophylaxis	
								100 (39.8–100.0, 4/4)	27.4 (17.6–39.1, 20/73)
								No Mold Prophylaxis	
								92.9 (6.1–99.8, 13/14)	73.1 (61.8–82.5, 57/78)
Imbert et al. 2016 (37)	Screening 2×/wk for IMD in patients with HM, HSCT, SOT, and other conditions	Proven or probable	60 (6 proven, 54 probable) / 881	Serum	1 mL	MagNA Pure Compact	LDT	Overall	
						kit (Roche)		71.7 (60.3–83.1, 43/60)	98.8 (98.1–99.5, 870/881)
								Mold Prophylaxis	
								70.8 (48.9–87.4, 17/24)	NP
								No Mold Prophylaxis	
								72.2 (54.8–85.8, 26/36)	NP

Loeffler et al. 2017 (13)	Screening 2×/wk for IMD in pediatric patients with HSCT	Probable	27-Apr	Serum and occasionally plasma	1 mL	QIAamp UltraSens virus kit (Qiagen)	LDT	100 (39.8–100, 4/4)	63% (42.4–80.6,17/27)
Seo et al. 2021 (38)	Suspected IMD in HM, BMT, SOT, and other conditions	Proven or probable	46 (13 proven, 33 probable) / 34	Plasma	0.2 mL	QIAamp DNA MiniKit (Qiagen)	LDT	54.3 (40.2–67.9, 25/46)	94.1 (80.9–98.4, 32/34)
Senchyna et al. 2021 (39)	Case control study	Proven or probable	25 (NP) / 107	Plasma	2 mL	Maxwell	LDT	56.3 (33.2–79.9, 9/16)	97.2 (92.0–99.4, 104/107)
						RSC ccfDNA Plasma Kit (Promega)			
Imbert et al. 2023 (40)	Suspected IMD in high-risk patients	Proven, probable , putative, CAPA	39 (2 proven, 19 probable, 4 putative, 14 CAPA) / 705	Serum	1 mL	Viral NA plasma extraction kit (Roche)	MycoGENIE	Overall	
								64.1 (48.4–77.3, 25/39)	98.6 (97.4–99.2, 695/705)
								Non-CAPA	
								84.0 (63.9–95.5, 21/25)	NP
								CAPA	
								28.6 (8.4–58.1, 4/14)	NP
Mah et al. 2023 (18)	Suspected IMD in HM, HSCT, SOT, and other conditions	Proven or probable	43 (15 proven, 31 probable) / 130	Plasma	4 mL	Maxwell	LDT	Overall	
						RSC ccfDNA Plasma Kit (Promega)		86.0 (72.7–93.4, 37/43)	93.1 (87.4–96.3, 121/130)
								Mold Prophylaxis	
								84.6 (57.7–95.7, 11/13)	95.7 (85.8–98.8, 45/47)
								New Diagnosis of IA	
								93.5 (80.9–98.5, 40/43)	93.1 (87.4–97.3, 121/130)
								HM/HSCT	
								92.0 (81.4–100, 23/25)	91.8 (82.2–96.5, 56/61)
								SOT/Other	
								76.5 (50.1–93.2, 13/17)	93.6 (82.5–98.7, 44/47)

^*a*^ IMD, invasive mold disease; HSCT, hematopoietic stem cell transplant; BMT, bone marrow transplantation; SOT, solid organ transplantation; HM, hematological malignancy; NP, not provided; LDT, laboratory developed test; CAPA, COVID-19-associated pulmonary aspergillosis; wk, week, EORTC/ MSGERC, European Organization for Research and Treatment of Cancer and the Mycoses Study Group Education and Research Consortium; CI, confidence interval. Sensitivities and specificities were compared using the Chi Square test.

**TABLE 2:** Clinical and assay characteristics and performance of Mucorales cell-free DNA PCR reported in published studies^*a*^

Study, Year (reference)	Patient population	EORTC/MSGERC Category for IMD cases	Number of cases (category) / controls	Specimen type	Sample volume	Extraction method	PCR assay (LDT vs commercial)	Sensitivity % (95% CI, n/N)	Specificity % (95% CI, n/N)
Legrand et al.2016(41)	Screening 2×/wk for IMD in patients with severe burns	Proven or probable	10 (6 proven, 4 probable) / 67	Plasma	1 mL	Qiasymphony DSP Virus/Pathogen kit (Qiagen)	LDT	100 (69.2–100, 10/10)	100 (94.6–100, 67/67)
Millon et al. 2016 (23)	Mucormycosis cases in HM, SOT, and other conditions	Proven or probable	44 (25 proven, 19 probable) / 0	Serum	1 mL	MagNA Pure Compact nucleic acid isolation kit I (Roche)	LDT	Overall	
								81.8 (67.3–91.8, 36/44)	ND
								Optimized Conditions	
								91.9 (78.1–98.3, 34/37)	ND
Springer et al. 2016 (42)	Screening 1–2×/wk for IMD in patients with HM and HSCT	Proven or probable	5 (NP) / 13	Serum	1 mL	QIAamp UltraSens virus kit (Qiagen)	LDT	100 (47.8–100, 5/5)	84.6 (54.6–98.1, 11/13)
Senchyna et al. 2021 (39)	Case control study	Proven or probable	12 (NP) / 113	Plasma	2 mL	Maxwell RSC ccfDNA Plasma Kit (Promega)	LDT	91.7 (62.5–100, 11/12)	98.2 (93.8–99.8, 111/113)
										Seo et al. 2021 (38)	Suspected IMD in HM, BMT, SOT, and other conditions	Proven or probable	21 (17 proven, 4 probable) / 59	Plasma	NP	QIAamp DNA MiniKit (Qiagen)	LDT	57.1 (36.6–75.5, 12/21)	76.3 (64.0–85.3, 45/59)
Millon et al. 2022 (43)	Suspected IMD in high-risk patients	Proven or probable	27 (20 proven, 7 probable) / 205	Serum	1 mL	Kits on QIAsymphony (Qiagen), Nucleic Acid Isolation Kit I (Roche), and NucliSENS easyMag (Biomerieux)	LDT	85.2 (66.3–95.8, 23/27)	89.8 (84.8–93.5, 184/205)
Imbert et al. 2023 (37)	Suspected IMD in high-risk patients	Proven, probable, or putative	20 (15 proven, 31 probable) / 724	Serum	1 mL	Viral NA plasma extraction kit (Roche)	MycoGENIE	80.0 (58.4–91.9, 16/20)	99.5 (98.6–99.8, 720/724)
Mah et al. 2025 (24)	Suspected IMD in HM, HSCT, SOT, and other conditions	Proven or probable	47 (34 proven, 13 probable) / 170	Plasma	4 mL	Maxwell	LDT	Overall	
						RSC ccfDNA Plasma Kit (Promega)		85.1 (71.7–93.8, 40/47)	92.9 (88.0–96.3, 158/170)
								Immunosuppressed	
								92.1 (78.6–98.3, 35/38)	93.9 (88.8–97.2, 139/148)
								Not Immunosuppressed	
								55.6 (21.2–86.3, 5/9)	86.4 (82.5–98.7, 19/22)
								Mold Prophylaxis	
								92.9 (76.5–99.1, 26/28)	93.0 (83.0–98.1, 53/57)
								HM/HSCT	
								92.3 (74.9–99.1, 24/26)	92.0 (84.1–96.7, 80/87)
								SOT/Other	
								91.7 (61.5–99.8, 11/12)	93.6 (82.5–98.7, 44/47)

Pfister et al. 2025 (44)	Suspected IMD in HM, HSCT, severe burns, and other conditions	Proven, probable, or PCR-only	27 (NP) / NP	Serum	NP	NP	MycoGENIE	77.8 (57.7–91.4 ,21/27)	ND

^*a*^ IMD, invasive mold disease; HSCT, hematopoietic stem cell transplant; BMT, bone marrow transplantation; SOT, solid organ transplantation; HM, hematological malignancy; NP, not provided; ND, not done; LDT, laboratory developed test; wk, week, EORTC/MSGERC, European Organization for Research and Treatment of Cancer and the Mycoses Study Group Education and Research Consortium; CI, confidence interval. Sensitivities and specificities were compared using the Chi Square test.

## INVASIVE ASPERGILLOSIS

**Fig 1:**
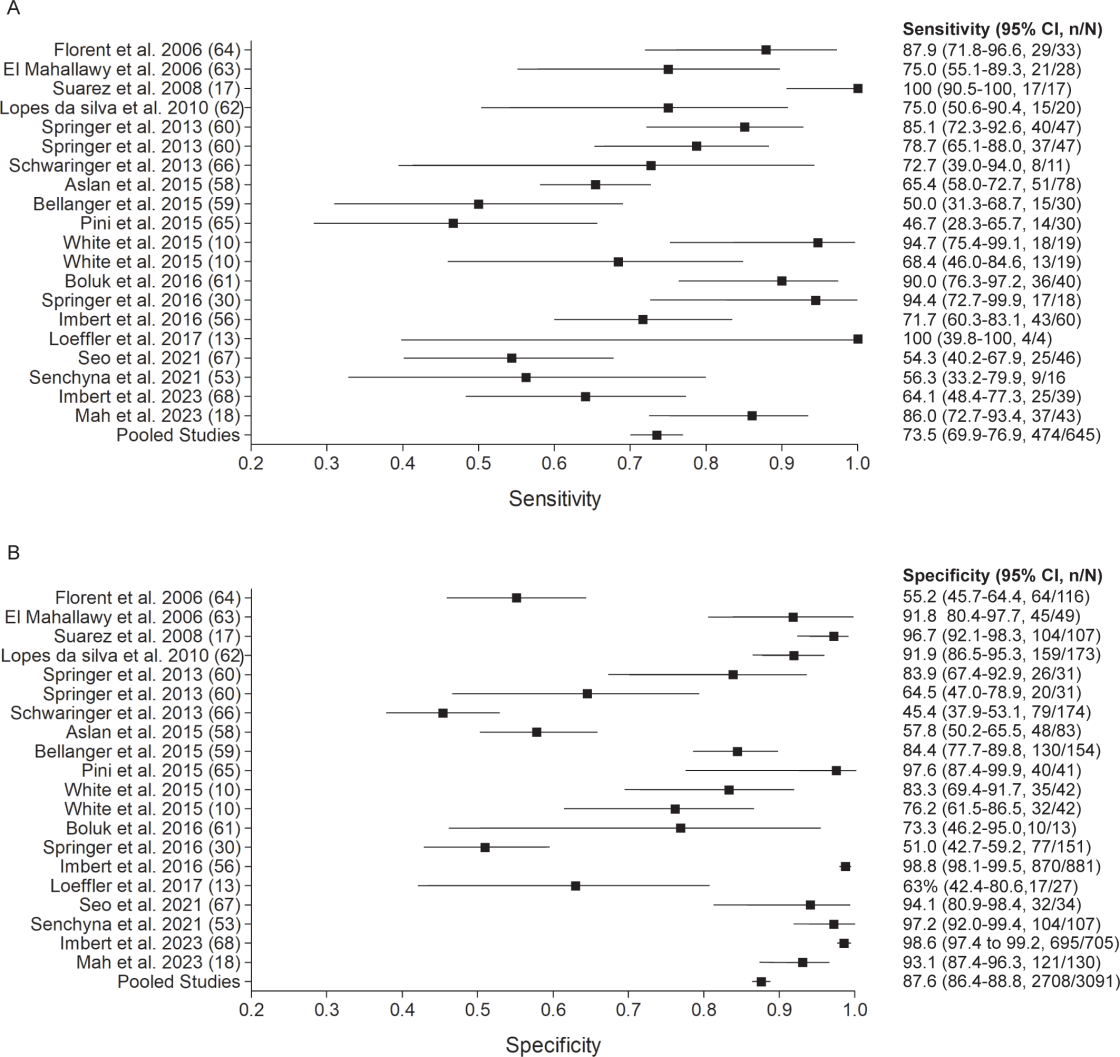
Forest plots showing sensitivity and specificity of *Aspergillus* cell-free DNA PCR reported in published studies investigating the diagnosis of IA. Studies are ordered by year of publication. The first author and reference number are shown. Pooled values are shown at the bottom. The squares and horizontal lines correspond to the reported or calculated sensitivity (**A**) and specificity (**B**) and their corresponding 95% confidence interval (CI).

In published studies investigating patients with IA, *Aspergillus* cfDNA PCR on plasma or serum had an overall pooled sensitivity and specificity of 73.5% (95% CI 69.9–76.9) and 87.6% (95% CI 86.4–88.8), respectively (Fig. 1; Table S1). There was a wide range in sensitivities (50.0–100) and specificities (45.4–98.8), which may be due to differences in pre-analytical variables, including testing indication and patient population, and analytical factors. A significantly higher sensitivity was noted in studies screening for IA in high-risk asymptomatic patients vs. testing symptomatic patients suspected of IA (80.2% [95% CI 74.4–85.2] vs. 68.7% [95% CI 62.9–74.1]) and between laboratory-developed tests and commercial assays (76.0% [95% CI 71.8–79.8] vs. 67.4% [95% CI 60.2–74.0]) (Table S1). However, there was a trade-off between sensitivity and specificity where a higher specificity was noted in studies testing symptomatic patients (87.5% [95% CI 84.1–90.4] vs. 70.1% [95% CI 67.1–73.0]) and commercial assays (94.2 [95% CI 92.4–95.7] vs. 85.1% [95% CI 83.6–86.6]) (Table S1). No significant differences were noted in pooled sensitivities in patients on and off mold prophylaxis (78.0% [95% CI 62.4–89.4] vs. 78.0% [95% CI 64.0–88.5]) and for serum vs. plasma (73.9% [95% CI 69.9–77.6]) vs. 71.8% (95% CI 63.0–79.5]), although the specificities were significantly higher in patients not on mold prophylaxis (73.1% [95% CI 61.8–82.5] vs. 54.2% [95% CI 44.8–63.3]) and in plasma (93.3% [95% CI 89.9–95.8] vs. 87.0 [95% CI 85.788.2], respectively) (Table S1).

In the future, using optimized pre-analytical and analytical variables, including plasma as the sample type and a large sample volume for extraction, will be imperative for studies evaluating the accuracy of *Aspergillus* cfDNA PCR (10, 18, 42). A recent study evaluating the clinical accuracy of a pre-analytically optimized *Aspergillus* plasma cfDNA PCR assay reported an overall sensitivity of 86.0% and specificity of 93.1%, and a sensitivity of 93.0% in patients with a new diagnosis of IA (18). The highest accuracy was reported in hematological malignancies (HM) and hematopoietic stem cell transplant (HSCT) patients (sensitivity 92.0%, specificity 91.8%). A modest sensitivity was reported in other immunosuppressed populations, including solid organ transplantation patients (sensitivity 76.5%, specificity 92.6%), which suggests *Aspergillus* plasma cfDNA may still be useful in non-neutropenic patients (18). Furthermore, unlike prior studies, which discouraged the usage of *Aspergillus* cfDNA PCR in patients on mold prophylaxis due to poor specificities (36, 45–47), this recent study demonstrated a high specificity (95.7%) in patients on mold prophylaxis (18).

As the performance of *Aspergillus* cfDNA PCR improves with pre-analytical and analytical optimization, it will be essential to understand the role of conventional non-invasive biomarkers in the diagnosis of IA. Only one study has directly compared the accuracy of *Aspergillus* plasma cfDNA PCR with serum GM for the diagnosis of IA (18). *Aspergillus* plasma cfDNA PCR was shown to have a significantly higher sensitivity (86.0% vs. 63.0%, respectively) in patients with proven or probable IA, but similar specificities in patients with no IA compared with serum GM (18). Combining plasma cfDNA PCR with serum GM improved the sensitivity of cfDNA PCR from 86.0% to 93.5% in all patients, and to 100% in patients with new diagnoses of proven and probable IA (18). Further studies are needed to determine whether the availability of an accurate *Aspergillus* cfDNA PCR assay limits the utility and actionability of GM.

## MUCORMYCOSIS

**Fig 2:**
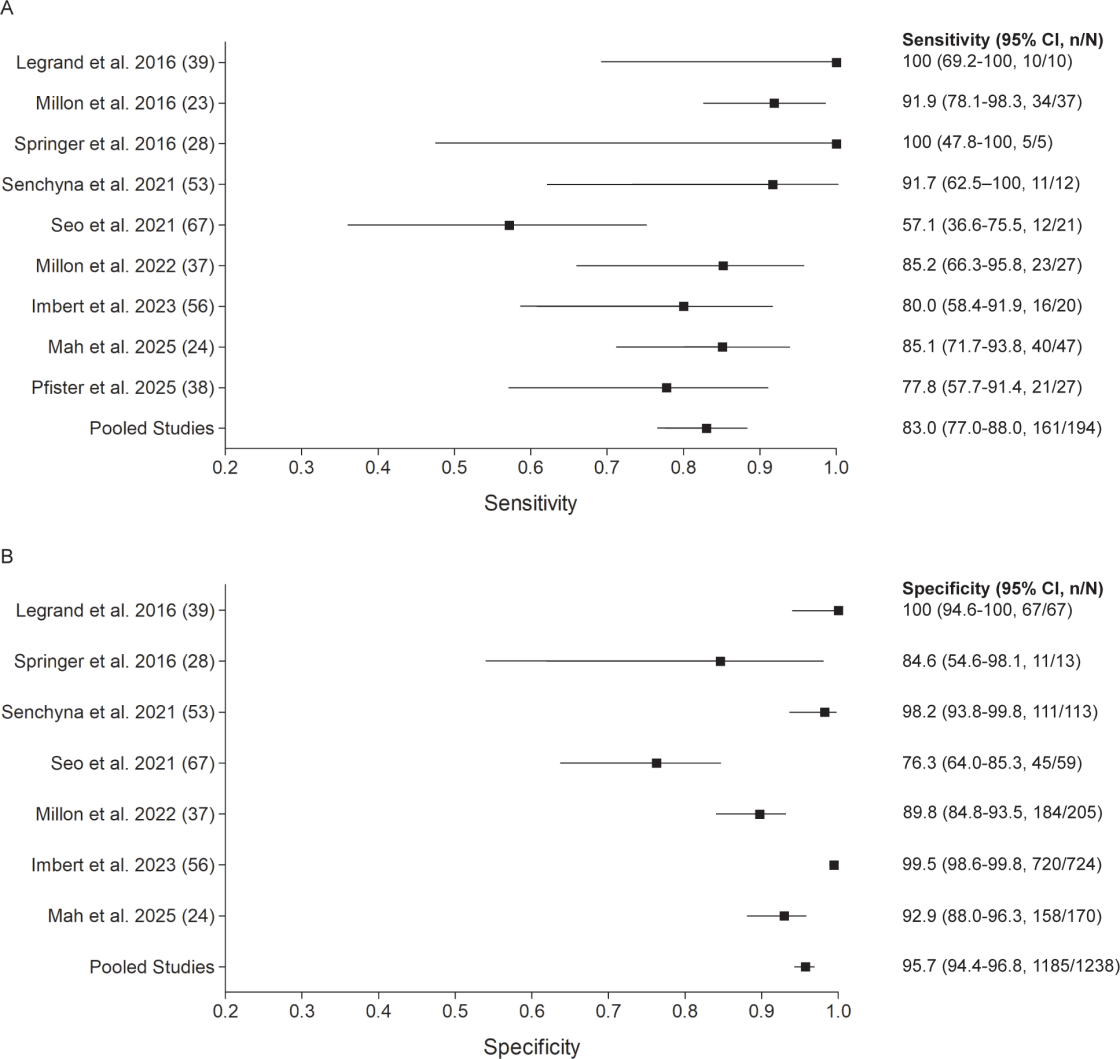
Forest plots showing sensitivity and specificity of Mucorales cell-free DNA PCR reported in published studies investigating the diagnosis of mucormycosis. Studies are ordered by year of publication. The first author and reference number are shown. Pooled values are shown at the bottom. The squares and horizontal lines correspond to the reported or calculated sensitivity (**A**) and specificity (**B**) and their corresponding 95% CI (CI).

Non-invasive cfDNA PCR is particularly attractive for the diagnosis of mucormycosis, given that a serum biomarker, such as GM and BDG, does not exist for Mucorales agents (48–51). A number of cfDNA PCR assays have been reported for the diagnosis of invasive mucormycosis (11, 19, 23, 41, 43, 44). These assays target the most prevalent genera of Mucorales, including *Mucor*, *Rhizopus*, *Rhizomucor*, and, in some assays, *Lichthemia*, based on the local epidemiology of this agent (23, 43, 52, 53). Overall, Mucorales cfDNA PCR on plasma or serum had a pooled sensitivity and specificity of 83.0% (95% CI 77.0–88.0) and 95.7% (95% CI 94.4–96.8), respectively (Fig. 2; Table S2). A wide range in sensitivities (57.1–100) and specificities (76.3–100) was observed, which is likely attributed to differences in pre-analytical and analytical variables. No significant differences were noted in pooled sensitivities and specificities in studies screening for mucormycosis vs. those testing symptomatic patients (sensitivity, 100% [95% CI 78.2–100] vs. 81.6% [95% CI 75.1–87.0]; specificity, 97.5% [91.3–99.7] vs. 95.6 [94.3–96.7] (Table S2). Although no significant differences were noted in pooled sensitivities for serum vs. plasma (85.3% [95% CI 77.6–91.2] vs. 79.5% [95% CI 68.8–87.8]) and between laboratory-developed tests and commercial assays (84.4% [95% CI 77.5–89.8] vs. 78.7% [95% CI 64.3–89.3]), the specificities were significantly higher for serum (97.1% [95% CI 95.9–98.1] vs. 91.2% [95% CI 87.4–94.2]) and for commercial assays (99.4% [95% CI 98.6–99.9] vs. 90.5% [95% CI 87.6–92.9], respectively (Table S2).

Studies have also shown a difference in sensitivity for Mucorales cfDNA PCR in immunosuppressed patients vs. non-immunosuppressed patients (54). A pre-analytically optimized Mucorales plasma cfDNA PCR assay reported a sensitivity and specificity of 92.1% (95% CI 78.6–98.3%) and 93.9% (95% CI 88.8–97.2%) respectively, in immunosuppressed patients, and 55.6% (95% CI 21.2–86.3%) and 86.4% (95% CI 82.5–98.7%), respectively, in non-immunosuppressed patients (24). A higher sensitivity in immunocompromised patients vs. non-immunosuppressed patients was also reported in a study using Mucorales serum cfDNA PCR (87.2% vs. 40.0%) (23). Furthermore, in the former study, Mucorales plasma cfDNA PCR retained its sensitivity in patients on mold-active prophylaxis, with a sensitivity of 92.6% (76.5–99.1%), thus supporting its use in patients with breakthrough infections (24).

## PNEUMOCYSTIS JIROVECII

*Pneumocystis jirovecii* plasma cfDNA PCR may also assist in non-invasive diagnosis of *P. jirovecii* pneumonia (PJP) when bronchoalveolar lavage fluid (BAL) or induced sputum cannot be obtained in clinically unstable patients (55, 56). Earlier studies investigating the accuracy of *P. jirovecii* cfDNA PCR reported conflicting sensitivities (57–61). In a more recent study, using a pre-analytically optimized assay, *P. jirovecii* plasma cfDNA PCR was shown to have a sensitivity and specificity of 100% and 93.4%, respectively, in proven PJP cases (62). The assay was less accurate in proven or probable cases, with a sensitivity and specificity of 48.6% and 99.1%, respectively (62). When comparing *P. jirovecii* plasma cfDNA PCR to BDG, both assays had a similar sensitivity in patients with proven PJP (BDG sensitivity 100%) and patients with proven or probable PJP (BDG sensitivity 60.0%). However, the specificity of *P. jirovecii* plasma cfDNA PCR was significantly higher compared to BDG in possible and no PJP patients (99.1% vs. 66.7%). Thus, if these results are reproduced in other studies, *P. jirovecii* plasma cfDNA PCR could replace BDG as a more specific but equally sensitive non-invasive diagnostic in patients with suspected PJP.

## DIAGNOSTIC ALGORITHMS

**Fig 3:**
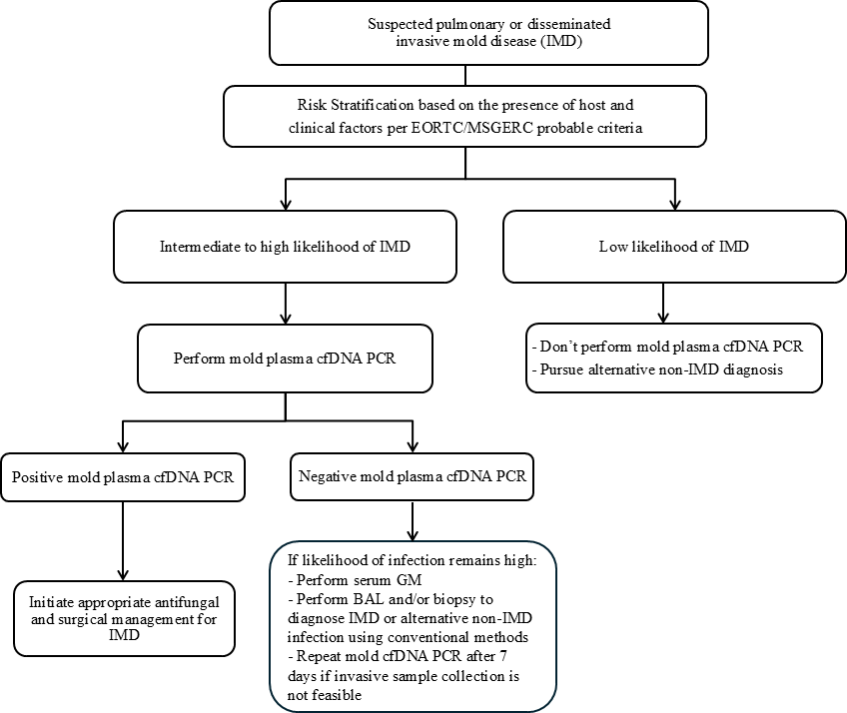
Diagnostic algorithm for IMD incorporating non-invasive mold plasma cfDNA PCR. IMD; EORTC/MSGERC; cfDNA; GM; BAL.

Given the high sensitivity of *Aspergillus* and Mucorales cfDNA PCR, a natural progression of this testing modality is to determine whether non-invasive plasma cfDNA PCR can replace invasive fungal testing on specimens collected via bronchoscopy and biopsy. A recent study evaluated the potential of a mold plasma cfDNA PCR (targeting *Aspergillus* species, Mucorales agents, *Fusarium* species, *Scedosporium* species, and *Lomentospora prolificans*) as a standalone test by investigating the concordance of non-invasive mold plasma cfDNA PCR with invasive testing on tissue and BAL tested with conventional fungal diagnostics (19). After adjudication of discordant cases based on the European Organization for Research and Treatment of Cancer and the Mycoses Study Group Education and Research Consortium (EORTC/MSGERC) case definitions, the mold plasma cfDNA PCR had a concordance of 88.5% with invasive specimen test results (19, 63). The discordance analysis showed that 64.7% of proven IFD cases with negative cfDNA PCR consisted of invasive fungal sinusitis cases in diabetic patients and superficial limb infections. The authors pointed out that in such cases, prompt surgical intervention should be the standard of care for both therapeutic and diagnostic purposes, and that diagnosis should not be contingent upon non-invasive testing with cfDNA PCR. Furthermore, the authors argued that some of the discordant results may have been due to false culture results. Among probable IFD cases with negative mold cfDNA PCR, 29.2% only had 1 or 2 fungal colonies growing in the BAL cultures, which could have represented colonization, contamination, or transient spore inhalation instead of true infection. Lastly, discordant cases with positive cfDNA PCR but negative BAL cultures, which made up 37.5% of discordant cases, could have been due to the limited sensitivity of fungal culture vs. false-positive results (Demir et al., Unpublished data). Given the findings of this study and other studies on the high accuracy of *Aspergillus* and Mucorales cfDNA PCR, a practical diagnostic algorithm for invasive mold disease (IMD) could start with non-invasive mold plasma cfDNA PCR in patients with intermediate to high likelihood of disseminated and pulmonary IMD followed by non-invasive serum GM testing and invasive testing samples such as BAL and tissue in patients with negative cfDNA PCR results who still have a high likelihood of infection and can undergo an invasive procedure (Fig. 3). This approach addresses a limitation of cfDNA PCR in that it cannot detect off-panel molds. The adoption of non-invasive mold cfDNA PCR could improve healthcare efficiency and quality by reducing the reliance on routine invasive specimen collection for the diagnosis of IFD, especially in unstable immunocompromised patients who have contraindications for invasive sampling.

## CLINICAL IMPACT AND PROGNOSTIC VALUE

Fungal cfDNA PCR has the ability to provide results days earlier compared with conventional diagnostic testing and thus allow for earlier initiation of optimized antifungal therapy and improved outcomes (10, 23, 43). In a prospective study, Mucorales serum cfDNA PCR was positive a median of 4 days prior to histopathological or microbiological diagnosis (10, 23, 43). Similarly, *Aspergillus* plasma cfDNA PCR was positive 16.8 days (range 0–28) before a diagnosis of proven or probable IA was made with conventional methods (10). In a prospective study of patients with suspected IFD, fungal cfDNA PCR was shown to have a positive impact on clinical management in 36.2% of patients with positive results (39). In a before-and-after study, twice-weekly screening of hematology patients with Mucorales serum cfDNA PCR was associated with a 48% lower one-month all-cause mortality rate (64).

Beyond the utility of cfDNA PCR for diagnosis of IMD, studies have shown Mucorales and *Aspergillus* cfDNA PCR can also have a prognostic value. Mucorales and *Aspergillus* cfDNA PCR baseline cycle thresholds have been shown to correlate with survival outcomes (65). Similarly, baseline fungal burden based on serum cfDNA PCR has been shown to correlate with patient survival (37). Additionally, for mucormycosis, repeated positive cfDNA despite antifungal initiation has been associated with worse survival compared to those who reverted to a negative cfDNA result (23, 43, 65). Thus, the prognostic information provided by mold cfDNA PCR may allow for identification of patients at risk of poor outcome who may benefit from alternative therapies.

## DIAGNOSTIC STEWARDSHIP

Although fungal cfDNA PCR can play a crucial role in establishing a diagnosis of IFD earlier in the course of disease, thus improving clinical management and patient outcome, there is still a need for diagnostic stewardship to improve the predictive value of the test and to reduce overutilization of resources (10, 23, 39, 43, 64). Follow-up mold plasma cfDNA PCR testing was reported to rarely generate a positive result considered clinically significant in the first week after an initial negative result (66). Similarly, the yield of repeating dimorphic fungi plasma cfDNA PCR was low over a 4-week interval following a negative result at baseline (66). Thus, if reproduced in other studies, it is reasonable to limit repeating fungal cfDNA PCR following a negative result to improve resource utilization and prevent false positive results.

## SUMMARY

Fungal cfDNA PCR has been shown to be highly sensitive and specific for the diagnosis of IA and mucormycosis in patients with proven and probable IFD. Further studies are needed to measure the accuracy of cfDNA PCR for the diagnosis of IFD caused by other agents, such as *Candida* spp., *Fusarium* spp., *Scedosporium* spp., *Lomentospora prolificans*, *P. jirovecii*, and dimorphic fungi. Prospective studies are needed to investigate pre-analytical and analytical variables using clinical samples and determine which variables have the biggest impact on fungal cfDNA PCR accuracy. Further studies are needed to determine the role of serial testing for the purpose of treatment monitoring. Current challenges of fungal cfDNA PCR include their limited availability, as they are currently only offered as laboratory-developed tests (LDTs) in some countries. However, given the recent court ruling against the regulation of LDTs by the FDA, more laboratories in the U.S. are expected to start offering fungal cfDNA PCR assays as LDTs. Regardless of this, future needs include the commercialization of cartridge-based assays on fully automated instruments for rapid, near-patient testing. Such advances are likely to increase the utilization of fungal cfDNA PCR and improve patient outcomes.

## SUPPLEMENTAL MATERIAL

Supplemental tables jcm.01236-24-s0001.xlsx Tables S1 and S2.

ASM does not own the copyrights to Supplemental Material that may be linked to, or accessed through, an article. The authors have granted ASM a non-exclusive, world-wide license to publish the Supplemental Material files. Please contact the corresponding author directly for reuse.
